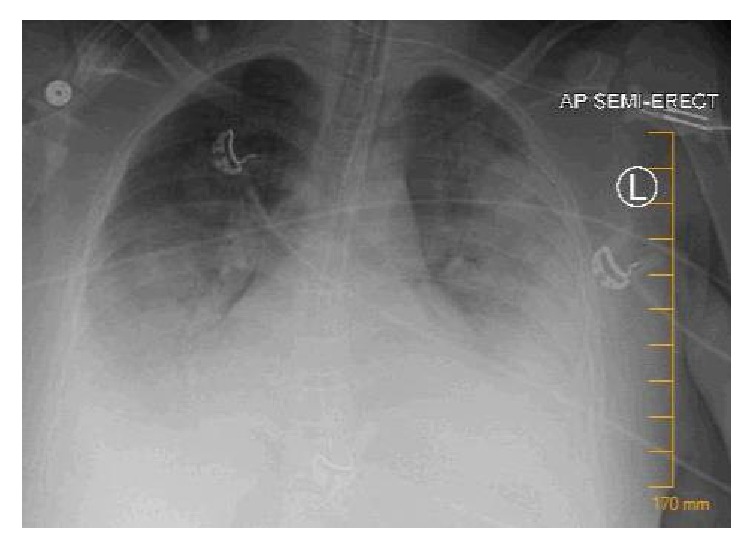# Acute Chest Syndrome Progressing to ARDS in a Patient of 25-Week Gestation

**DOI:** 10.1155/2018/4243569

**Published:** 2018-01-30

**Authors:** Jefferson Chambers, Nichole Smith, Matthew Sehring, Subramanyam Chittivelu

**Affiliations:** ^1^University of Illinois College of Medicine at Peoria, Peoria, IL, USA; ^2^Illinois Lung Institute, Peoria, IL, USA

## Abstract

Acute chest syndrome is a complication of sickle cell disease and represents the highest cause of mortality in those afflicted with the disorder. Pregnancy represents an increased risk for complications of sickle cell disease in both the mother and fetus. We present a case of a 20-year-old patient with known sickle cell disease who was at 25-week gestation and developed acute chest syndrome refractory to conventional therapies and requiring emergency cesarean section. Following delivery, the patient developed acute respiratory distress syndrome (ARDS) requiring extracorporeal membrane oxygenation (ECMO). The patient and infant eventually made full recoveries. This case highlights the importance of aggressive management of ACS and careful monitoring in a pregnant patient.

## 1. Introduction

Acute chest syndrome (ACS) is a complication of sickle cell crisis (SSC) defined by radiographic densities, fever, and respiratory symptoms [1]. About 50 percent of patients with sickle cell disease will experience an episode of ACS sometime in their life, which carries a mortality of approximately 4 percent. We present a difficult case of ACS in a pregnant patient causing fetal distress prompting emergency cesarean section and eventual extracorporeal membrane oxygenation due to acute respiratory distress syndrome (ARDS).

## 2. Case Presentation

A 20-year-old female with a past medical history significant for sickle cell disease (SSD) (unknown genotype) and current pregnancy (G1P0; 25 weeks, 3 days) was admitted for acute sickle cell pain crisis.

**Figure 1:**
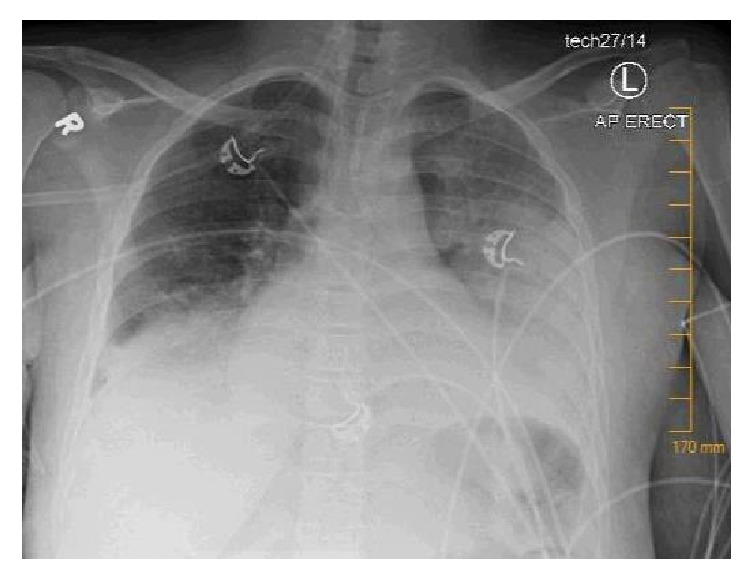


Shortly after admission, the patient experienced fever to 104 degrees Fahrenheit, tachycardia to 122 beats per minute, and tachypnea with a respiratory rate of 58 breaths per minute. She remained normotensive with an oxygen saturation of 96%. Physical exam was remarkable for moderate distress and diffuse bilateral pulmonary rales. Laboratory testing was significant for white blood cell count of 53.3 G/L, and differential included 88% neutrophils, 4.5% lymphocytes, 3.3 G/L monocytes, 0.15 G/L basophils, 3+ poikilocytosis, 2+ polychromasia, 1+ Howell-Jolly bodies, and 1+ vacuolated polymorphonuclear lymphocytes with target and sickle cells present. Hemoglobin was 8.1 g/dL, with reticulocyte index of 21.8%. Chest X-ray demonstrated bilateral airspace opacifications, worse on the left (Figure 1).

She was initially treated with exchange red blood cell transfusions, noninvasive positive pressure ventilation, IV fluid resuscitation, broad spectrum antibiotics, and IV pain medications for suspected acute chest syndrome.

Shortly after admission, fetal distress was noted which was attributed to intermittent hypoxia and narcotic effects. The patient developed progressive respiratory acidosis and required emergent fetal delivery and intubation with mechanical ventilation. Despite continued management, the patient required increasing positive end expiratory pressure (PEEP) and fraction of inspired oxygen (FiO2) up to 14 and 100%, respectively. She further required manual PEEP maneuvers without improvement. Repeat chest X-ray showed progression of pulmonary opacities (Figure 2) and the decision was made to pursue venovenous (VV) extracorporeal membrane oxygenation (ECMO) due to severe acute respiratory distress syndrome (ARDS).

Following cannulation of the right internal jugular vein, ECMO was initiated and ventilator settings were weaned. Over the next several days, she had marked clinical improvement and was successfully extubated and decannulated. She continued to receive supportive blood transfusions to keep HgbS concentration below 30% and hemoglobin above 10, in total requiring 7 units of packed red blood cells. She received a total of 10-day antibiotic therapy and was discharged home on day 12. At present, both mother and son are doing well.

## 3. Discussion

Acute chest syndrome is a sickle cell crisis involving the pulmonary vasculature and tissues. It is characterized by pulmonary densities on chest imaging, fever, respiratory distress, hypoxemia, and chest pain [1]. Acute chest syndrome is the number one cause of mortality among patients with SSD [2]. The mainstay of treatment for acute chest syndrome involves RBC exchange transfusions to remove the irreversibly sickled HgbS and replace it with normal hemoglobin. This reduces the total HgbS concentration, lowering potential for vaso-occlusion. Goals for exchange transfusions include a HgbS concentration of less than 30 percent.

Pregnancy complicates SSD and results in an increased risk of acute pain crises and ACS.

Over 55% of women with SSD will have at least one pain crisis during their pregnancy [3]. Other complications in SSD during pregnancy include hypertension, preeclampsia, venous thromboembolism, and increased risk of cesarean section. Risks to the fetus include intrauterine growth restriction and fetal demise due to placental hypoperfusion [4]. Some have proposed that pregnant women undergo regular blood transfusions in an attempt to prevent acute pain crises. However, in one retrospective study, prophylactic transfusions in pregnant patients with SSD did not improve SSD-related pregnancy complications [5]. More recently, a prospective study demonstrated improved outcomes for mother and baby with prophylactic transfusions [6]. This remains a controversial topic and there are no clear recommendations for or against prophylactic exchange transfusions in pregnancy.

Overall, management of a pregnant patient with ACS remains similar to that of a nonpregnant patient. Antibiotics, pain management, aggressive fluid resuscitation, and, in severe cases, exchange transfusions remain the mainstay of therapy. Potential complications during pregnancy include placental vasoconstriction secondary to opioids and direct placental damage secondary to active sickling [3]. These two factors place the fetus in grave danger of placental insufficiency during an episode of SSC or ACS. Thus, continuous fetal monitoring with EFM is paramount in the management of a patient in acute crisis. EFM and a multidisciplinary team approach in our patient allowed for timely detection of fetal distress leading to intervention which ultimately saved the life of the infant.

ACS progressing to ARDS remains a rare, but life threatening condition. As with any cause of ARDS, early intervention is of utmost importance. One of these interventions is ECMO which provides a means for reversal of hypoxemia and hypercarbia. ECMO will not fix the underlying pulmonary process; however, it provides supportive management, allowing time for resolution of ARDS in situations when mechanical ventilation is inadequate or there is concern for ongoing lung injury secondary to ventilation. Indications for ECMO include severe ARDS plus either a PaO2/FiO2 of less than 80 or pH of less than 7.25 for 3 or more hours despite attempted interventions [7].

## 4. Conclusion

In a gravid patient with severe acute chest syndrome, aggressive oxygenation and exchange transfusions must be performed in order to ensure safety of the fetus and prevent the progression into or worsening of ARDS. Close collaboration of a multidisciplinary team is recommended to achieve the best outcomes for both the mother and fetus. In cases where ACS progresses to ARDS, ECMO and treatment of the underlying problem may improve outcomes.

## Figures and Tables

**Figure 2:**